# Frequency of hemorrhage after tooth extraction in patients treated with a direct oral anticoagulant: A multicenter cross-sectional study

**DOI:** 10.1371/journal.pone.0266011

**Published:** 2022-04-07

**Authors:** Iwabuchi Hiroshi, Sawai Y. Natsuko, Imai Yutaka, Shirakawa Masayori, Nakao Hiroyuki, Imai Hirohisa

**Affiliations:** 1 Kanagawa Dental University, Yokosuka-shi, Japan; 2 Department of Dentistry and Oral Surgery International University of Health and Welfare Hospital, Nasushiobara-shi, Japan; 3 Department of Education Planning, Faculty of Dentistry, Kanagawa Dental University, Yokosuka-shi, Japan; 4 Dokkyo Medical University School of Medicine, Shimotsuga-gun, Japan; 5 First Department of Oral and Maxillofacial Surgery, Meikai University School of Dentistry, Saitama, Japan; 6 Faculty of Nursing, Miyazaki Prefectural Nursing University, Miyazaki, Japan; 7 Department of Medical and Pharmaceutical Community Healthcare, Graduate School of Medicine, University of Tokyo, Tokyo, Japan; Inselspital, Bern University Hospital, SWITZERLAND

## Abstract

**Objective:**

Few studies have compared the frequency of hemorrhages after tooth extraction between patients taking direct oral anticoagulants (DOACs) and those taking warfarin or no anticoagulants. Further, the effects of the timing of DOAC administration and tooth extraction on the frequency of post-extraction hemorrhage have not been demonstrated. Therefore, we compared the frequency of post-extraction hemorrhages in patients in these different conditions and examined the effects of the timing of DOAC administration and tooth extraction on the frequency.

**Design:**

Prospective multicenter study

**Setting:**

Eighty-six Japanese hospitals.

**Participants:**

In total, 182 teeth extracted from 145 individuals (119 teeth from adult males) receiving dabigatran and 88 teeth from individuals (62 teeth from adult males) receiving rivaroxaban were included.

**Intervention:**

Tooth extraction was followed by a 7-day observational period between November 1, 2008 and December 31, 2015. Dabigatran was administered twice daily; rivaroxaban was administered once a day.

**Primary outcome measure:**

Hemorrhage after tooth extraction.

**Results:**

The frequency of hemorrhage after tooth extraction was 1.65%, 3.41%, and 3.63% in those treated with dabigatran, rivaroxaban, and warfarin, respectively, and 0.39% in those who did not receive anticoagulants. Hemorrhages after tooth extraction were significantly higher in the rivaroxaban group than in patients who did not receive anticoagulants (P = 0.008). These frequencies did not differ significantly in the dabigatran and rivaroxaban groups compared to the warfarin group (P = 0.221 and P = 1.000, respectively).

**Conclusions:**

The frequency of hemorrhaging after tooth extraction appeared to be similar in patients receiving continuous dabigatran or rivaroxaban and in those receiving continuous warfarin.

## Introduction

In a multicenter study [1] initiated in November 2008 to validate the safety of tooth extraction in Japanese patients treated with anticoagulant therapy, we reported that the difference in the frequency of hemorrhage after extraction between warfarin-treated and non-warfarin groups was 3.24% (95% confidence interval: 1.49–4.99%). The risk factors for hemorrhage after extraction in those receiving warfarin treatment were age, prothrombin time–international normalized ratio (PT-INR), and acute inflammation at the extraction site [1]. Warfarin remains a commonly used anticoagulant agent; however, direct oral anticoagulants (DOACs) have recently been developed, and the use of these drugs has increased rapidly.

Although it is desirable that tooth extractions in patients taking DOACs are performed under continuous administration of the drug, as in patients taking warfarin, it is reported that bleeding after tooth extraction under continuous administration of DOAC was more frequent compared to patients who underwent tooth extraction without taking antithrombotic drugs [2]. On the other hand, some reports [3, 4] suggest that the incidence of bleeding events after tooth extraction in patients taking DOAC was not different from that of patients taking warfarin. In addition, many reports indicate that simple tooth extraction in patients receiving dabigatran etexilate methanesulfonate (hereafter abbreviated as dabigatran) can be performed with continuous administration of the drug without any problems if appropriate local hemostasis treatment is used in combination [5–10]. Reports also indicate that even patients being treated with rivaroxaban can undergo tooth extraction while being continuously administered the drug and receiving appropriate local hemostasis treatments in combination, as long as there are no abnormalities with their kidney function and there is no risk of bleeding [8, 11]. However, there is yet to be enough research on the safety of tooth extraction in patients taking DOAC, and most past reports have been retrospective studies and single-site studies involving a small number of subjects.

Therefore, we conducted a multicenter prospective study to evaluate the safety of tooth extraction in patients who are being continuously administered a DOAC, which is a case we expect to see more often in the future. In this study, we investigated the frequency of bleeding events after tooth extraction under continuous DOAC administration and compared this to continuous warfarin administration or patients not receiving any anticoagulant treatment. In addition, we examined the effects that the timing of DOAC administration and the timing of tooth extraction have on the frequency of bleeding events after tooth extraction.

## Methods

### Study design

We prospectively examined the presence or absence of bleeding events after tooth extraction while under continuous warfarin administration, continuous DOAC administration, or non-administration of anticoagulants during the period from confirming primary hemostasis to 7 days after tooth extraction (suture removal). The DOACs used in the study were dabigatran (Prazaxa; Nippon Boehringer Ingelheim Co., Ltd., Tokyo Japan) and rivaroxaban (Xarelto; Bayer Yakuhin, Ltd., Tokyo Japan).

### Study duration and subjects

This multicenter study on the safety of tooth extraction while receiving anticoagulant therapy was conducted in the departments of oral surgery of 68 hospitals in Japan from November 1, 2008 to December 31, 2015. The cases collected were those with simple extraction of permanent dentition conducted under continuous administration of DOAC, continuous administration of warfarin, or non-administration of anticoagulants in the collaborating sites during the case-collection period that met all of the inclusion criteria and violated none of the exclusion criteria. Patients receiving warfarin and patients not receiving any anticoagulant treatments were enrolled between November 1, 2008, and March 31, 2010 [1], while patients receiving DOAC treatments were enrolled between January 1, 2013 and December 31, 2015. A simple extraction was defined as an extraction procedure that did not involve detachment or eversion of the mucoperiosteal flap and shaving of the alveolar bone. The inclusion criteria were the following: patients who underwent primary hemostasis using a local hemostatic technique recommended by the guidelines for extraction in Japanese patients treated with anticoagulant therapy [12], age ≥20 years at the time of extraction, treated by a surgeon with ≥3 years of experience, extraction time ≤15 min, platelet count of 100,000 or higher during the 7 days after tooth extraction, and PT-INR of <3.0 during the 7 days after tooth extraction for patients taking warfarin who could undergo tooth extraction without reducing the dose or discontinuing warfarin administration. Patients with poor hemostatic function were excluded.

### Observation items

The items observed included the presence or absence of hemorrhages after tooth extraction, patient age/sex, extraction site, extraction method (forceps only, elevators only, forceps + elevators), underlying condition that led to tooth extraction, use/non-use of antiplatelet drugs, PT-INR during the 7 days after tooth extraction (only for patients taking warfarin), presence or absence of comorbidities that may affect hemostasis and the name of the condition, use/non-use of vasoconstrictor drugs, use/non-use of vasoconstrictor-added local anesthetic, use/non-use of inferior alveolar nerve block, gingival inflammation at the extraction site (none, mild, moderate, severe), defective granulation tissue in the extraction socket (none, mild, moderate, severe), presence or absence of acute inflammatory conditions at the extraction site, and presence or absence of post-extraction infections [1]. In patients administered DOACs, we also investigated the extraction timing (9 am–12 pm, 12–3 pm, 3–6 pm), activated partial thromboplastin time (APTT), serum creatinine concentration within the 3 months prior to extraction, and the administration time (morning or evening) for subjects treated with dabigatran. The prothrombin time (PT) and serum creatinine concentration within the 3 months prior to extraction were recorded for subjects treated with rivaroxaban.

### Hemostatic technique

Wound margins were sutured, and an extraction socket was filled with oxidized cellulose or a gelatin sponge in subjects treated with a DOAC or warfarin. No local or systemic hemostatic drug or hemostatic splint was used before confirming primary hemostasis. If multiple teeth were extracted at the same time, we checked for the presence or absence of bleeding after extraction for each tooth removed; for patients that were administered warfarin or DOAC, hemostasis treatments were provided by suturing and filling the extraction socket with oxidized cellulose or gelatin sponge. For patients who were not administered any anticoagulant, the surgeon chose the most suitable hemostatic technique according to the condition of each individual patient.

### Use of drugs

The use of local anesthetics, including vasoconstrictors (epinephrine and felypressin), was allowed at their normal doses. As a general rule, patients who received warfarin were administered CFPN-PI or CDTR-PI, a penicillin or cephem antibiotic that is said to have less interaction with antibacterial agents; patients with penicillin allergies were administered CAM. We decided not to limit the administration of analgesics such as acetaminophen or non-steroidal anti-inflammatory drugs (NSAIDs) and cyclooxygenase-2 (COX-2) inhibitors as long as they were used at their respective normal doses [1].

### Confirmation of hemostasis

After tooth extraction, hemostasis was performed by applying pressure with gauze. Hemostasis was confirmed upon releasing pressure after less than 30 minutes of compression. After primary hemostasis, patients were checked for post-extraction hemorrhaging on the day after tooth extraction. Post-extraction hemorrhaging in patients was monitored by instructing patients to visit the clinic if there were any bleeding events after the day following tooth extraction. Patients were monitored for 7 days after tooth extraction to see if there was any post-extraction hemorrhage [1].

### Examination of hemorrhage after extraction

If hemorrhage occurred after an extraction in patients who received continuous DOAC administration, the following conditions were measured as soon as possible: bleeding conditions, blood pressure, APPT, and serum creatinine concentrations were determined in the dabigatran group, and PT and serum creatinine concentrations were measured in the rivaroxaban group.

### Assessment of hemorrhage after extraction

In this study, hemorrhage events occurring in the follow-up period were classified into one of the following five grades: 0, no hemorrhage; 1, excessive blood clotting in the socket, no treatment required; 2–1, hemostasis achieved by compressing the wound longer than 30 min; 2–2, oozing hemorrhage observed on or after the next day of the procedure, with hemostasis being achieved by simple compression; 3, hemorrhage requiring treatment other than wound compression, such as application of compression brace and/or electrotome coagulation. As described previously [1], grade 2–2 and higher events were regarded as clinically significant and were defined as hemorrhage post-extraction in this study. Bleeding evaluation after tooth extraction was performed by a dentist with at least 3 years of clinical experience (similar to that of the surgeon).

### Statistical analysis

Data aggregation was carried out for each extracted tooth and for each patient. Data were collected separately for each extracted tooth. Therefore, when more than one tooth was simultaneously extracted from one subject, plural case cards (based on the number of extracted teeth) contained the same subject’s background information. Differences in the frequency of hemorrhage after extraction in those treated with dabigatran, rivaroxaban, warfarin, or no anticoagulation therapy were determined. Multivariate analysis was performed to identify the risk factors for hemorrhage after extraction in the DOAC groups. Using hemorrhage after extraction as an objective variable and known risk factors as explanatory variables, logistic regression was performed to determine the odds ratios, P-values, and 95% confidence intervals after excluding the effects of multiple explanatory variables on a single objective variable. Variables with P-values < 0.2 in the univariate analysis were used in the multivariate analysis. All statistical analyses were performed using Statistical Package for the Social Sciences (SPSS) v.23.0 (IBM SPSS Japan, Tokyo, Japan). P-values <0.05 were considered statistically significant.

### Ethical considerations

All participants received a full explanation of the objectives of the study before providing verbal informed consent. Personal information was collected only during the observation period and was deleted after the 7-day observation period ended. This study was approved by the institutional ethics committee of the National Hospital Organization Tochigi Medical Center (approval number 26–2).

### Patient characteristics

We considered problematic hemorrhages after tooth extraction in patients taking DOAC. Life-threatening hemorrhaging after a tooth extraction is highly unlikely. Furthermore, minor bleeding not requiring treatment is not problematic. Thus, we examined the frequency of only the clinically problematic hemorrhages that followed tooth extraction.

## Results

### Characteristics of the subjects

Of the 278 teeth extracted from subjects treated with DOACs, 8 were excluded due to protocol deviations or missing records. The remaining 270 teeth were extracted from 145 subjects. The below results in patients who were not receiving warfarin or anticoagulants have been reported previously [1]. According to analysis by each tooth extracted, the total number of teeth extracted from patients that were administered warfarin continuously was 496 teeth, with a mean patient age of 70.3 ± 10.9 years old; 320 teeth (64.5%) were extracted from men, and 176 teeth (35.5%) were extracted from women. The total number of teeth extracted from patients not administered an anticoagulant was 2,321, with the mean patient age being 60.4 ± 18.3 years old; 1,126 teeth (48.5%) were extracted from men and 1,195 teeth (51.5%) were extracted from women [1]. The dabigatran group (average age: 69.4 ± 8.21 years) included 182 teeth, 119 (65.4%) of which were extracted from males and 63 (34.6%) of which were extracted from females. The rivaroxaban group (average age: 70.1 ± 9.51 years) included 88 teeth, 62 (70.5%) of which were extracted from males and 26 (29.5%) of which were extracted from females (Table 1). According to analysis by each patient, there were 361 patients who were continuously administered warfarin and 2,146 patients who were not given any anticoagulant [1]. There were 94 patients (64.8%) who were continuously administered dabigatran, with a mean age of 69.7 ± 8.59 years; 69 patients (73.4%) were men and 25 patients (26.6%) were women. There were 51 patients (35.2%) who were continuously administered rivaroxaban, with a mean age of 70.6 ± 10.2 years old; 29 patients (56.9%) were men and 22 patients (43.1%) were women.

**Table 1:** Patient characteristics.

	Dabigatran	Rivaroxaban	Warfarin	None
	n = 182	n = 88	n = 496	n = 2321
**Age**				
< 65 years	35 (19.2)	25 (28.4)	124 (25.0)	1131 (48.7)
≥ 65 years	147 (80.8)	63 (71.6)	372 (75.0)	1190 (51.3)
**Sex**				
Male	119 (65.4)	62 (70.5)	320 (64.5)	1126 (48.5)
Female	63 (34.6)	26 (29.5)	176 (35.5)	1195 (51.5)
**Type**				
Foretooth	61 (33.5)	32 (36.4)	132 (26.6)	663 (28.6)
Premolar	49 (26.9)	22 (25.0)	127 (25.6)	552 (23.8)
Molar	72 (39.6)	34 (36.8)	237 (47.8)	1106 (47.7)
**Instrument**				
Forceps only	52 (28.6)	32 (36.4)	171 (34.5)	604 (26.0)
Elevators only	98 (53.8)	40 (45.5)	226 (45.6)	919 (39.6)
Forceps and elevators	32 (17.6)	16 (18.2)	99 (20.0)	798 (34.4)
**Cause for extraction**				
Periodontitis	87 (47.8)	33 (37.5)	160 (32.3)	533 (23.0)
Caries	33 (18.1)	18 (20.5)	87 (17.5)	477 (20.6)
Apical periodontitis	47 (25.8)	23 (26.1)	219 (44.2)	1002 (43.2)
Wisdom tooth pericoronitis	8 (4.4)	5 (5.7)	23 (4.6)	192 (8.3)
Other	7 (3.8)	9 (10.2)	7 (1.4)	117 (5.0)
**Status of antiplatelet therapy**				
Yes	22 (12.1)	30 (34.1)	122 (24.6)	433 (18.7)
**Use of local anesthetics containing vasoconstrictors**				
Yes	178(97.8)	87 (98.9)	491 (99.0)	2265 (97.6)
**Use of mandibular foramen conduction anesthesia**				
Yes	6 (3.3)	4 (4.5)	14 (2.8)	82 (3.5)
**Comorbidity possibly influencing hemostasis**				
Hypertension	75 (41.2)	42 (47.1)	267 (53.8)	715 (30.8)
Diabetes mellitus	22 (12.1)	17 (19.3)	89 (17.9)	267 (11.5)
Chronic hepatitis	2 (1.1)	8 (9.1)	21 (4.2)	110 (4.7)
Other	18 (9.9)	11 (12.5)	68 (13.7)	212 (9.1)
**History of acute inflammation at extraction site**				
Yes	27 (14.8)	30 (34.1)	216 (43.5)	829 (35.7)
**Gingival inflammation at extraction site**				
None	50 (27.5)	21 (23.9)	298 (60.1)	1266 (54.5)
Mild	103 (56.6)	44 (50.0)	95 (19.2)	264 (11.4)
Moderate	26 (14.3)	17 (19.3)	13 (2.6)	38 (1.6)
Severe	3 (1.6)	6 (6.8)	90 (18.1)	753 (32.4)
**Abnormal granulation tissue in extraction socket**				
None	50 (27.5)	23 (26.1)	87 (17.5)	818 (35.2)
Minimal	75 (41.2)	36 (40.9)	230 (46.4)	917 (39.5)
Moderate	28 (15.4)	20 (22.7)	134 (27.0)	441 (19.0)
Considerable	29 (15.9)	9 (10.2)	45 (9.1)	145 (6.2)
**Severity of post-extraction bleeding**				
G1	2 (1.1)	4 (4.5)	9 (1.8)	29 (1.2)
G2-1	1 (0.5)	0	8 (1.6)	11 (0.5)
G2-2	1 (0.5)	1 (1.1)	1 (0.2)	8 (0.3)
G3	2 (1.1)	2 (2.3)	17 (3.4)	1 (0.04)
Total	6 (3.3)	7 (8.0)	35 (7.1)	49 (2.1)
G2-2+G3	3 (1.6)	3 (3.4)	18 (3.6)	9 (0.34)

The values presented indicate the number of patients and percentage among the group.

### Frequency of hemorrhage after extraction

According to the analysis by each tooth, the frequency of post-extraction hemorrhages was 3.63% in cases with continuously administered warfarin and 0.39% in cases not administered any anticoagulant [1]. Hemorrhages after extraction occurred in three cases each in the dabigatran group (1.6%) and the rivaroxaban group (3.4%) (Table 2). No hemorrhages occurred after extractions of multiple teeth. According to the analysis by each patient, the frequency of post-extraction hemorrhaging was 2.77% in patients continuously administered warfarin and 4.2% in patients not administered any anticoagulant [1]. Post-extraction hemorrhages occurred in 3 patients (3.2%) who were continuously administered dabigatran and in 3 patients (5.9%) continuously administered rivaroxaban (Table 2).

**Table 2:** Incidences of clinically significant post-extraction bleeding and their difference.

		Incidence over total numbers of teeth extracted				
		95% CI				
	Post-extraction bleeding incidences (%)	LOWER (%)	UPPER (%)	vs. No anticoagulants	vs. Warfarin	vs. Dabigatran
				P value	P value	P value
No anticoagulants	0.39	0.18	0.73			
Warfarin	3.63	2.16	5.67	<0.001		
Dabigatran	1.65	0.34	4.74	0.051	0.221	
Rivaraxaban	3.41	0.71	9.64	0.008	1.000	0.395
Fisher’s exact test						
		Incidence over total numbers of study subjects				
		95% CI				
	Post-extraction bleeding incidences (%)	LOWER (%)	UPPER (%)	vs. No anticoagulants	vs. Warfarin	vs. Dabigatran
				P value	P value	P value
No anticoagulants	0.39	0.16%	0.81%			
Warfarin	2.77	1.34%	5.04%	<0.001		
Dabigatran	3.19	0.66%	9.04%	0.011	0.737	
Rivaraxaban	5.88	1.23%	16.24%	0.002	0.209	0.425
Fisher’s exact test						

CI: Confidence interval

### Comparison of the frequency of hemorrhage among all groups

According to the analysis by each tooth, the frequencies of hemorrhaging after extraction did not differ significantly between the group that received no anticoagulant and the groups that received dabigatran (0.39% vs. 1.65%, respectively; P = 0.051) or rivaroxaban (0.39% vs. 3.41%, respectively; P = 0.008). Similarly, there was no significant difference in the frequencies of hemorrhaging after extraction in the dabigatran and rivaroxaban groups compared to the warfarin group (P = 0.221 and P = 1.000, respectively). Similarly, the frequency of hemorrhaging after extraction did not differ significantly between the dabigatran and rivaroxaban groups (P = 0.395) (Table 2).

According to the analysis by each patient, the frequencies of post-extraction hemorrhaging in patients continuously administered dabigatran and patients not given any anticoagulant were 3.19% and 0.39%, respectively, with a significant difference between the two groups (P = 0.011). The frequencies of post-extraction hemorrhaging in patients continuously administered rivaroxaban and patients not given any anticoagulant were 5.88% and 0.39%, respectively, which were significantly different (P = 0.002). There were no differences in the frequencies of post-extraction hemorrhaging between patients who were continuously administered warfarin and patients who were continuously administered dabigatran (P = 0.737), or patients who were continuously administered rivaroxaban (P = 0.209). In addition, there was no difference in the frequencies of post-extraction hemorrhaging between patients who were continuously administered dabigatran and patients who were continuously administered rivaroxaban (P = 0.425) (Table 2).

### Risk factors for hemorrhage after extraction in the dabigatran and rivaroxaban groups

In the dabigatran group, no risk factor for hemorrhage after extraction was detected in either the univariate or multivariate analyses (Tables 3 and 4). In the rivaroxaban group, P-values < 0.2 were observed in the univariate analysis for the use of nerve blocks to the inferior alveolus, as well as for gingival inflammation at the extraction site. In the multivariate analysis that was performed using these factors as explanatory variables, the extraction technique (OR: 25.808, P = 0.039) and the use of nerve block injections to the inferior alveolus (OR: 31.606, P = 0.044) emerged as risk factors for hemorrhage after extraction (Tables 3 and 5).

**Table 3:** Univariate analysis of post-extraction bleeding events.

	Dabigatran					Rivaroxaban				
	Incidence of post-extraction bleeding (%)		95%CI			Incidence of post-extraction bleeding (%)		95%CI		
		OR	Lower	Upper	P-value		OR	Lower	Upper	P-value
**Age**										
< 65 years	0	1 (Ref)				0	1 (Ref)			
≥ 65 years	2.0					4.8				
**Sex**										
Male	1.7	1 (Ref)				4.8	1 (Ref)			
Female	1.6	0.944	0.084	10.612	0.962	0.0				
**Type**										
Foretooth or premolar	1.8	1 (Ref)				1.9	1 (Ref)			
Molar	1.4	0.761	0.068	8.545	0.825	5.9	3.312	0.289	38.013	0.336
**Instrument**										
Forceps only	2	1 (Ref)				1.4	1 (Ref)			
Elevators alone or with forceps	0					12.5	10.143	0.860	119.666	0.066
**Cause of extraction**										
Caries, apical periodontitis, or other	2.3	1 (Ref)				2.0	1 (Ref)			
Periodontitis or wisdom tooth pericoronitis	1.1	2.722	0.238	31.192	0.421	5.3	2.722	0.238	31.192	0.421
**Status of antiplatelet therapy**										
No	0	1 (Ref)				3.4	1 (Ref)			
Yes	1.7					3.3	0.966	0.084	11.099	0.978
**Use of vasoconstrictors**										
No	4.1	1 (Ref)				0	1 (Ref)			
Yes	0					3.4				
**Use of mandibular foramen conduction anesthesia**										
No	1.7	1 (Ref)				2.4	1 (Ref)			
Yes	0					25.0	13.6667	0.953	195.894	0.054
**Comorbidity possibly influencing hemostasis**										
No	1.4	1 (Ref)				0	1 (Ref)			
Yes	1.9	1.390	0.124	15.621	0.789	4.5				
**History of acute inflammation at extraction site**										
No	1.9	1 (Ref)				1.7	1 (Ref)			
Yes	0					6.9	4.071	0.3540	46.838	0.260
**Gingival inflammation at extraction site**										
None to mild	1.7	1 (Ref)				1.5	1 (Ref)			
Moderate to severe	6.7	2.696	0.236	30.754	0.424	8.7	6.095	0.526	70.672	0.148
**Abnormal granulation tissue in extraction socket**										
None to minimal	1.6	1 (Ref)				1.7	1 (Ref)			
Moderate to considerable	1.8	1.098	0.0980	12.365	0.940	6.9	4.296	0.3730	49.464	0.242

OR: Odds ratio, CI: Confidence interval

**Table 4:** Multivariate analysis of risk factors for post-extraction bleeding (dabigatran).

	Incidence of post-extraction bleeding (%)				
		OR	95%CI		P-value
			Lower	Upper	
**Sex**					
Male	1.7	1 (Ref)			
Female	1.6	0.957	0.080	11.41	0.867
**Type**					
Foretooth or premolar	1.8	1 (Ref)			
Molar	1.4	0.676	0.053	8.618	0.763
**Cause of extraction**					
Caries, apical periodontitis, or other	2.3	1 (Ref)			
Periodontitis or wisdom tooth pericoronitis	1.1	0.439	0.036	5.369	0.520
**Comorbidity possibly influencing hemostasis**					
No	1.4	1 (Ref)			
Yes	1.9	1.425	0.117	17.378	0.781
**Gingival inflammation at extraction site**					
None to mild	1.7	1 (Ref)			
Moderate to severe	6.7	3.261	0.185	57.656	0.420
**Abnormal granulation tissue in extraction socket**					
None to minimal	1.6	1 (Ref)			
Moderate to considerable	1.8	0.778	0.042	14.563	0.867

OR: Odds ratio

**Table 5:** Multivariate analysis of risk factors for post-extraction bleeding events (rivaroxaban).

	Incidence of post-extraction bleeding (%)	OR	95%CI		P-value
			Lower	Upper	
**Instrument**					
Forceps only	1.4	1 (Ref)			
Elevators alone or with forceps	12.5	25.808	1.174	567.253	0.039
**Use of mandibular foramen conduction anesthesia**					
No	2.4	1 (Ref)			
Yes	25.0	31.606	1.095	991.876	0.044
**Gingival inflammation at extraction site**					
None to mild	1.5	1 (Ref)			
Moderate to severe	8.7	19.339	0.791	472.607	0.069

OR: Odds ratio, CI: Confidence interval

### DOAC administration and tooth extraction times in subjects with hemorrhage after extraction

In the dabigatran group, hemorrhage after extraction occurred in one subject treated between 9 am and 12 pm, in two subjects treated between 12 and 3 pm, and in no subjects treated between 3 and 6 pm. In the rivaroxaban group, hemorrhage after extraction only occurred in subjects treated between 9 am and 12 pm (Table 6).

**Table 6:** Incidence of clinically significant post-extraction bleeding according to the timings of direct oral anticoagulant administration and tooth extraction.

Time of extraction	Rivaroxaban				Dabigatran	
	Taken in the morning	Post-extraction bleeding	Taken in the evening	Post-extraction bleeding	Taken in the morning and evening	Post-extraction bleeding
	n (%)		n (%)		n (%)	
9–12 AM	18 (31.0)	3	3 (75.0)	0	35 (32.1)	1
12–3 PM	28 (48.3)	0	1 (25.0)	0	55 (50.5)	2
3–6 PM	12 (20.7)	0	0	0	19 (17.4)	0

Values are n or n (%).

## Discussion

The use of DOACs has rapidly increased as an alternative to warfarin treatment. DOACs are classified as direct thrombin inhibitors, such as dabigatran, and direct factor Xa inhibitors, including rivaroxaban, apixaban, and edoxaban. No difference in the frequency of hemorrhage after tooth extraction has been noted for DOAC- and warfarin-treated patients [3, 4]; however, in a comparative study of tooth extractions in DOAC-treated patients and those not treated with anticoagulants, hemorrhages occurred more frequently after extraction in the DOAC group [2]. There have already been many reports of studies related to post-extraction hemorrhage in patients who were continuously administered warfarin, which indicated that serious hemorrhage after tooth extraction while continuously receiving warfarin can be avoided as long as appropriate local hemostatic technique is used. However, there are few such reports for DOAC. As such, we conducted this study thinking that if we are able to demonstrate that there is no difference between the frequencies of post-extraction hemorrhage between patients continuously administered DOAC and patients continuously administered warfarin, it would be possible to elucidate that the likelihood of serious bleeding after tooth extraction under continuous DOAC administration would be low.

The following three outcome measures were investigated in this nationwide, multicenter study: (i) the frequency of hemorrhages after extraction in DOAC-treated patients; (ii) differences in the frequency of hemorrhages after extraction between those receiving anticoagulant treatment and those who were not receiving it; and (iii) risk factors associated with hemorrhages after extraction in those receiving DOAC treatment. According to the analysis by each tooth, we observed that the frequency of hemorrhages after extraction was 1.65% and 3.41% in those treated with dabigatran and rivaroxaban, respectively, with this difference being nonsignificant. Neither percentage differed significantly from that observed in patients treated with warfarin (3.63%) [1].

Similar to the results of the analysis stratified by tooth, the results of the analysis stratified by the patient showed that the prevalence of post-extraction hemorrhaging was higher in patients administered dabigatran and rivaroxaban than in those not administered any anticoagulant but not different from that of the patients continuously administered warfarin; the results were the same regardless of the stratification by tooth or patient. Here, we focused our analysis on analysis by tooth. This is because warfarin is easily affected by meals and drugs, and general patient factors are suspected as being involved in post-extraction hemorrhaging in patients receiving warfarin. However, as DOAC is not easily affected by factors such as meals, we thought that the involvement of the extraction-site condition in post-extraction hemorrhaging might be greater than the individual general patient factors. We believe that the results of our study support this and also demonstrate the validity of conducting the analysis by tooth rather than by patient.

The results of the multivariate analysis did not identify any significant factors associated with an increased risk of a hemorrhage after extraction in those receiving dabigatran. The extraction technique and nerve block injections to the inferior alveolus were detected as significant risk factors for hemorrhages after extraction in those receiving rivaroxaban. For warfarin, the risk factors for hemorrhages after extraction were age, administration of anticoagulants, PT-INR, and a history of acute inflammation at the extraction site [1]. To date, there has been no randomized, comparative study of anticoagulant use in patients treated with DOACs. The combined use of multiple anticoagulants in DOAC-treated patients did not increase the risk for hemorrhaging after extraction in our study. Other studies have shown that hemorrhages after tooth extraction [13] or surgical procedures [14] in patients treated with warfarin and other anticoagulant agents is rare; this was also true for DOAC treatment. One possible reason to explain why the extraction technique was detected as a significant risk factor in those treated with rivaroxaban could be that tissue damage occurred as a result of the extraction; however, it remains unclear why nerve block injection to the inferior alveolus was identified as a risk factor for hemorrhages.

Various large-scale studies have demonstrated that monitoring of anticoagulant activity is not necessary for dabigatran and rivaroxaban because these drugs exhibit predictable pharmacokinetics and pharmacodynamics and have broad therapeutic indices [15]. However, in DOAC-treated patients, the incidences of hemorrhagic complications and thromboembolism were similar to those in patients treated with warfarin, adding uncertainty about the need for monitoring anticoagulant activity. Therefore, we measured anticoagulant activity and serum creatinine concentrations in subjects who experienced a hemorrhage after tooth extraction. The blood dabigatran concentration is reflected by the diluted thrombin time; however, this is difficult to determine. Thus, we determined the APTT, the utility of which is described in the practical guide of the European Heart Rhythm Association [16]. PT measurement is also useful for evaluating anticoagulant activity following rivaroxaban administration [16]. Therefore, we also examined anticoagulant capacity and serum creatinine levels and evaluated their relation with hemorrhages after tooth extraction. However, the frequency of hemorrhages after tooth extraction was too low to detect an association between these variables.

For patients taking DOAC, van Diermen et al. [10] recommend performing tooth extraction from 1–3 hours after oral administration of DOAC onward, and practical guides from the European Heart Rhythm Association [13] recommend performing extraction once trough concentrations are reached; this is, at least 12 hours after DOAC administration. The different recommendations on the timing from oral administration to tooth extraction are thought to be due to differences in the time necessary to reach maximum serum concentrations (Tmax) between fasting and postprandial conditions. That said, all these reports consider Tmax as an important factor and encourage performing tooth extraction during trough concentrations. In the present study, we also examined the relationship between the DOAC administration and tooth extraction times and hemorrhage after tooth extraction, although due to the low frequency of hemorrhages after tooth extraction, we could not observe any significant association.

This is a long-term study. When this study was initiated, warfarin was the main anticoagulant used in Japan. Therefore, we first investigated patients treated with warfarin and started collecting data on those treated with DOACs after analysis. Furthermore, when data collection was started for those treated with DOACs, the number of patients taking DOACs was only about one-tenth of those taking warfarin; therefore, accumulation of cases took a lot of time. However, the selection criteria and hemostasis methods of the study subjects, evaluation of post-extraction bleeding, selection criteria of the surgeon, and background survey items have not changed consistently since the start of the study. In addition, it is unlikely that the hemostasis method, tooth extraction method, patient’s coagulation ability, or response to anticoagulants have changed significantly within a few years. Therefore, we believe that the study period has little effect on the frequency of post-extraction bleeding in patients taking anticoagulants, which is the main purpose of this study. Furthermore, this study did not exclude patients taking antiplatelet drugs. Therefore, the proportion of patients taking antiplatelet drugs among those who continued to receive rivaroxaban and warfarin was higher than that in patients who did not take anticoagulants or receive continuous doses of dabigatran, which may have affected the study results. However, the frequency of post-extraction bleeding in patients receiving continuous administration of dabigatran and rivaroxaban did not differ in the univariate analysis depending on whether they took antiplatelet drugs. This result implies that the proportion of patients taking antiplatelet drugs has little effect on the study results.

This was an observational study and the evaluator was not blinded. The lack of drug blinding may have been a bias in the tooth extraction method selection. Dabigatran was not identified as a risk factor in multivariate analysis, especially in terms of whether the extraction method poses a risk of post-extraction bleeding; however, for rivaroxaban, the use of elevators posed a risk of post-extraction bleeding. This may indicate that the frequency of post-extraction bleeding was lower than it should be had the surgeon arbitrarily avoided the use of elevators in rivaroxaban-administered cases. However, the usage rates of elevators were 71.4% in patients treated with dabigatran, 63.7% in those treated with rivaroxaban, and 65.6% in those treated with warfarin. Since there were no significant differences in the usage rates of elevators, it is unlikely that the surgeon arbitrarily avoided the use of elevators in those receiving rivaroxaban. Furthermore, since there are many participating facilities, there is a concern that the evaluation of post-extraction bleeding may vary depending on the evaluator. To reduce variability among evaluators, a dentist with at least 3 years of clinical experience was chosen as the evaluator, and the evaluation method was made to be as simple as possible. The definition of post-extraction bleeding (Grade 2–2 and higher events) was defined only when compression hemostasis was required the day after tooth extraction and when non-compression treatment was required to induce hemostasis. In addition, the evaluation method was sufficiently disseminated and discussed with the dentist in advance to reduce the variation in the evaluation.

## Conclusion

According to the analysis by each tooth, the frequencies of hemorrhages after tooth extraction were 1.65% in patients receiving dabigatran and 3.41% in those treated with rivaroxaban. In addition, the frequency of hemorrhages after tooth extraction was higher in patients receiving continuous warfarin than in patients not receiving anticoagulants and was similar to that of patients receiving continuous rivaroxaban. The frequency of hemorrhages after tooth extraction in patients receiving dabigatran was similar to that of patients not taking anticoagulants.

## Supporting information

S1 File (XLS)
