# Correction: Frequency of hemorrhage after tooth extraction in patients treated with a direct oral anticoagulant: A multicenter cross-sectional study

**DOI:** 10.1371/journal.pone.0303944

**Published:** 2024-05-15

**Authors:** Iwabuchi Hiroshi, Sawai Y. Natsuko, Imai Yutaka, Shirakawa Masayori, Nakao Hiroyuki, Imai Hirohisa

In the Author Contributions, Imai Yutaka should have not been listed as one of the persons who acquire funding but as one who wrote the paper.
